# Normoxic versus hyperoxic reperfusion: A retrospective study of ischemia–reperfusion injury in patients undergoing on-pump coronary artery bypass grafting

**DOI:** 10.1177/02676591261438387

**Published:** 2026-03-21

**Authors:** Youssef El Dsouki, Ignazio Condello, Orhan Eren Gunertem, Abducelil Yildirim, Serdar Gunaydin, Roberto Lorusso

**Affiliations:** 1 82246 Maastricht University Medical Centre MUMC, Maastricht, the Netherlands; 2 Cardiovascular Research Centre Maastricht CARIM, Maastricht, the Netherlands; 3 School of Medicine and Surgery, 19045 University of Insubria, Varese, Italy; 4 Department of Cardiovascular Surgery, Medical Park Hospital, YIH University, Ankara, Turkey

**Keywords:** normoxia, hyperoxia, cardiopulmonary bypass, myocardial injury, oxidative stress

## Abstract

**Background:**

Blood oxygen content and management during cardiopulmonary bypass (CPB), particularly during reperfusion phase after cross-clamping related ischemia, may influence systemic oxygen utilization, oxidative stress, and organ ischemia/reperfusion injury. These conditions may be highly relevant in patients undergoing coronary artery bypass grafting (CABG) due to coronary artery disease. The impact of a hyperoxia/normoxia strategy on metabolic efficiency, myocardial injury, and early clinical outcomes remains incompletely defined.

**Materials and methods:**

We conducted a retrospective study including 50 patients undergoing isolated elective CABG with CPB. Patients were stratified into a normoxia (N) group (PaO_2_ 90–130 mmHg; *n* = 25) and a hyperoxia (H) group (PaO_2_ >200 mmHg; *n* = 25). CPB trend parameters including PaO_2_, mixed venous oxygen saturation (SvO_2_), indexed oxygen extraction ratio, and lactate levels were analyzed intraoperatively. Myocardial injury was assessed by serial troponin measurements. Early postoperative left ventricular ejection fraction (LVEF), need for inotropic support, ICU and hospital length of stay, as well as biomarkers of oxidative stress and hypoxia-related pathways, were evaluated. Exploratory outcomes included lactate kinetics (0, 6, 12, and 24 h), acute kidney injury (KDIGO criteria), new-onset atrial fibrillation within 48 h, duration of mechanical ventilation, and 30-days major adverse cardiac events (MACEs).

**Results:**

Baseline, including preoperative LVEF, as well as operative characteristics, including types of grafts, modalities of revascularization (single vs jumpgraft) as well as cardiopulmonary bypass and aortic cross-clamp durations, were similar between groups. All grafts underwent flow assessment at the end of the operation to confirm the quality and appropriateness of perfusion. During CPB, indexed oxygen delivery was comparable; however, the hyperoxia group showed significantly higher arterial and mixed venous oxygen partial pressures, associated with a lower oxygen extraction ratio and higher lactate concentrations, indicating reduced metabolic efficiency. Troponin levels were significantly lower in the normoxia group during CPB and at 6, 12, and 24 h postoperatively, reflecting attenuated myocardial injury. Post-CPB LVEF was higher in the normoxia group (51.8 ± 8.9 vs 46.3 ± 10.1%, *p* = 0.041), accompanied by a reduced need for inotropic support (24% vs 56%, *p* = 0.021). Intensive care unit and hospital length of stay were significantly shorter in normoxic patients. Hyperoxic oxygenation was associated with significantly higher perioperative levels of hypoxia-inducible factor-1α, total oxidant status, and oxidative stress index, along with reduced total antioxidant status.

**Conclusions:**

In patients undergoing CABG with CPB, normoxia was associated with more efficient oxygen utilization, reduced oxidative stress, , attenuated perioperative myocardial injury, improved early postoperative ventricular function, and shorter ICU and hospital stay compared with hyperoxic oxygenation. These findings support a physiology-guided oxygenation strategy during cardiac surgery.

## Introduction

Coronary artery bypass grafting (CABG) performed with cardiopulmonary bypass (CPB) and aortic cross-clamping with cardioplegia-induced cardiac arrest inevitably exposes the myocardium to a period of global ischemia followed by reperfusion, a pathophysiological sequence that remains a major determinant of perioperative myocardial injury, early postoperative cardiac dysfunction, increased need for inotropic support, and prolonged intensive care unit (ICU) stay.^1,2^ Arterial oxygen management during CPB and early reperfusion represent predisposing factors to such adverse events and potentially modifiable components of this complex and deleterious process.^3^ In routine clinical practice, hyperoxic oxygenation is frequently adopted during CPB, often targeting arterial oxygen tensions greater than 200 mmHg, with the intent of maintaining a safety margin against hypoxemia.^4,5^ This approach is largely pragmatic, with no robust or largely agreed evidence-based knowledge, rather than physiology-guided and reflects current limitations in intraoperative monitoring. In most centers, arterial blood gas analysis performed intermittently at serial time intervals remains a primary tool to assess systemic oxygenation during CPB, although continuous monitoring of arterial oxygen saturation and, in contemporary platforms, continuous PaO_2_ measurement may also be available.^6^ However, real-time integration of these parameters into physiology-guided oxygen titration strategies is not uniformly implemented.^7^ At the same time, contemporary perfusion practice has increasingly embraced goal-directed perfusion (GDP) strategies aimed at optimizing systemic oxygen delivery. Maintaining an indexed oxygen delivery (DO_2_i) above critical thresholds, commonly identified around 280 mL/min/m^2^, has been associated with improved metabolic stability and reduced risk of organ dysfunction during CPB.^8^ However, even when adequate DO_2_i targets are achieved, arterial oxygen tension is often allowed to reach supra-physiological levels, highlighting a potential dissociation between oxygen delivery optimization and oxygenation strategy.^9^ Importantly, adequate or supra-normal global oxygen delivery does not necessarily translate into effective cellular oxygen utilization. Elevated PaO_2_ may paradoxically be accompanied by reduced oxygen extraction ratios, reflecting a mismatch between oxygen supply and consumption.^8^ This dissociation may lead to metabolic inefficiency or maladaptive changes with lactate accumulation, indicating a state of relative cellular dysoxia despite apparently sufficient systemic oxygenation, a phenomenon sometimes described as “luxury oxygenation.” Moreover, sustained exposure to supra-physiological oxygen tensions may exert detrimental biological effects through oxidative mechanisms. Hyperoxia is associated with increased generation of reactive oxygen species (ROS), which impair endothelial function and reduce nitric oxide bioavailability.^5^ Conversely, maintaining normoxia during CPB and reperfusion, while simultaneously ensuring adequate oxygen delivery through GDP strategies, may preserve a more favorable balance between oxygen supply and utilization.^9,10^ Therefore, the present retrospective study aimed to compare normoxia and hyperoxic oxygenation strategies during CPB in a limited patient series undergoing isolated elective CABG managed with a GDP approach targeting a DO_2_i ≥ 280 mL/min/m^2^. By integrating CPB oxygenation trends (PaO_2_, mixed venous oxygen saturation, oxygen extraction, and lactate kinetics), biomarkers of myocardial injury and oxidative stress, and early clinical outcomes including postoperative LVEF, inotropic support, and length of ICU and hospital stay, we sought to provide a comprehensive evaluation of the physiological and clinical consequences of oxygenation strategies during CPB and reperfusion.

## Materials and methods

### Study design and ethical approval

This retrospective study was conducted between August and Septmeber 2024. Ethical approval for the study was obtained from the University Institutional Review Board of K.Kale Nr 2024.11.22-2. All procedures were performed in accordance with institutional guidelines and the ethical standards outlined in the Declaration of Helsinki. Given the retrospective observational design and use of anonymized data, the Institutional Review Board waived the requirement for individual informed consent.

### Patient population

Fifty consecutive adult patients undergoing isolated CABG with CPB were included. To evaluate the impact of oxygenation strategy during CPB on myocardial injury, perfusion physiology, oxidative stress, and clinical outcomes, two chronologically consecutive care periods were examined. During the earlier period, CPB was managed conventionally using a roller pump–based extracorporeal circuit with traditional management of gas blender. Regulation of FiO_2_ and sweep gas flow relied exclusively on intermittent arterial blood gas (ABG) values, and practice routinely resulted in supraphysiological PaO_2_ values greater than 200 mmHg. Patients treated during this period formed the Hyperoxia Group (*n* = 25). In the later period, the center implemented a perfusion platform with integrated continuous gas monitoring. Continuous measurement and feedback-controlled adjustment of PaO_2_ and PaCO_2_ enabled real-time titration of gas flow and FiO_2_ to maintain PaO_2_ within physiological ranges (90–130 mmHg) during CPB. Patients treated using this system formed the Normoxia Group (*n* = 25). Aside from the monitoring platform change, surgical technique, perfusion targets, anesthesia, myocardial protection strategy, and postoperative clinical care remained unchanged between the two periods (Table 1).

**Table 1.** Study population and outcomes in Normoxia group versus Hyperoxia group.

CABG procedures (*n* = 50)	Normoxia group (mean ± std) (*n* = 25)	Hyperoxia group (mean ± std) (*n* = 25)	*p*. value
EF %	52.52 ± 9.45	56.46 ± 12.38	0.32
Priming (ml)	1300	1300	1.0
Lactate baseline (mg/dL)	1.22 ± 0.28	1.25 ± 0.30	0.71
Age (Years)	69.22 ± 4.05	67.41 ± 7.83	0.69
Weight (kg)	78.0 ± 9.22	76.26 ± 0.93	0.070
Height (cm)	165.57 ± 5.71	164.53 ± 8.61	0.78
BSA (m^2^)	1.82 ± 0.12	1.70 ± 0.12	0.31
BMI (kg/m^2^)	27.44 ± 3.13	27.27 ± 3.47	0.58
Calculated blood flow (l/min)	4.408.44 ± 283.67	4.449.6 ± 299.04	0.88
Bypass time (minutes)	79.0 ± 23.21	76.13 ± 18.17	0.34
Cross clamp time (minutes)	53.23 ± 13.55	59.14 ± 14.33	0.38
EF % after CPB	51.84 ± 8.92	46.27 ± 10.11	0.041
Need for inotropic support after CPB, *n* (%)	6 (24%)	14 (56%)	0.021
Mechanical ventilation time (hours)	12.26 ± 3.443	15.22 ± 2.15	0.22
ICU stay (days)	1.56 ± 1.18	2.23 ± 1.25	0.01
Hospital stay (days)	7.13 ± 1.08	9,21 ± 1.39	0.05
Sex (*n*) (Male/Female)	13/12	14/11	0.35

CPB, Cardiopulmonary Bypass; EF %, Ejection Fraction Percentage; BSA (m^2^), Body Surface Area in square meters; BMI (kg/m^2^), Body Mass Index in kg/m^2^; ICU Stay (days), Intensive Care Unit Stay in days; Hospital Stay (days).

### Inclusion and exclusion criteria

Patients ≥18 years of age undergoing isolated elective CABG requiring CPB and with available complete perioperative data were eligible. Patients undergoing urgent or emergency CABG, those with major comorbidities potentially affecting outcomes (severe renal insufficiency with creatinine clearance <30 mL/min, advanced hepatic dysfunction, systemic infection and chronic obstructive pulmonary Disease), patients with pre-existing systemic inflammatory or autoimmune disorders, individuals who experienced myocardial infarction or major cardiovascular events within the preceding 3 months, and patients with incomplete perioperative data or missing postoperative outcome documentation were excluded. No structured long-term follow-up beyond 30-days clinical outcomes was planned in this study.

### Anesthetic management

All patients received balanced general anesthesia. Induction consisted of intravenous propofol, midazolam, opioid analgesia, and neuromuscular blockade rocuronium (Esmeron®, Merck Sharp & Dohme, USA) or cisatracurium (Nimbex®, GlaxoSmithKline, United Kingdom), followed by controlled mechanical ventilation. Tidal volume and respiratory frequency were adjusted to maintain normocapnia (PaCO_2_ 35–45 mmHg) with FiO_2_ titrated to achieve arterial oxygen saturation exceeding 95%. Maintenance anesthesia consisted of inhaled volatile anesthetics or intravenous propofol infusion combined with opioid infusion titrated to hemodynamic response. Monitoring included invasive arterial pressure, pulse oximetry, end-tidal CO_2_, core temperature, urinary output, central venous pressure, and ECG. Near-infrared spectroscopy (NIRS) monitoring using a Masimo O3® system (Masimo Corporation, Irvine, CA, USA) was applied in all patients for continuous assessment of regional cerebral oxygen saturation. Transesophageal echocardiography was routinely used in all patients, while pulmonary artery catheterization was employed when clinically indicated.

### Anticoagulation and hemostasis

Anticoagulation was initiated before cannulation using intravenous unfractionated heparin at a dose of 300–400 IU/kg. Activated clotting time (ACT) was monitored at predefined intervals and maintained above 480 s during CPB. After CPB discontinuation and completion of surgical hemostasis, protamine sulfate was administered to reverse heparin in doses guided by ACT measurements. Conservative transfusion approaches, including intraoperative cell salvage and ultrafiltration, were utilized in accordance with institutional blood management practices.

### Surgical procedure and myocardial protection

All operations were performed through a median sternotomy. Following systemic heparinization, cannulation of the ascending aorta and right atrium was performed. CPB was instituted using normothermic systemic perfusion. Myocardial protection was achieved in all patients using intermittent isothermal blood cardioplegia according to the Calafiore protocol, delivered both antegrade and retrograde.^11^ Oxygenated arterial blood from the CPB circuit was mixed with potassium and magnesium delivered in an antegrade fashion into the aortic root… and in a retrograde fashion via the coronary sinus. An initial dose was administered until diastolic cardiac arrest was achieved, followed by maintenance doses at fixed 20-min intervals throughout the cross-clamp period. Additional doses were delivered at the discretion of the surgical team in the presence of electrical activity or myocardial contractility. Three-vessel coronary artery bypass grafting was performed in the majority of patients (92% in the Normoxia group and 88% in the Hyperoxia group), with left internal mammary artery grafting to the left anterior descending artery used in all patients. Revascularization of the marginal branch with the right internal mammary artery was achieved in 88% and 84% (*p* = NS) of patients in the Normoxia and Hyperoxia groups, respectively, while a saphenous vein graft to the posterior descending artery was performed in 92% and 88% (p = NS) of cases. Distal coronary anastomoses were performed during aortic cross-clamping, whereas proximal anastomoses were constructed after aortic unclamping using side-clamping. Standard de-airing maneuvers were performed before clamp removal. To minimize potential bias related to myocardial protection and graft performance, all anastomosed grafts were intraoperatively assessed using transit time flowmetry (TTFM). Graft quality was confirmed by satisfactory mean graft flow, pulsatility index, diastolic filling percentage, and backward flow values, all exceeding accepted cut-off thresholds, indicating optimal graft function. Intraoperative graft assessment was performed using transit time flowmetry (*VeriQ™, Medistim, Oslo, Norway*).^12^

### Cardiopulmonary bypass configuration and perfusion strategy

Arterial blood gas (ABG) samples were obtained at predefined time points: after initiation of CPB, every 20 min during CPB (corresponding to cardioplegia intervals), immediately before aortic unclamping, and at CPB weaning. In the Hyperoxia Group, ABGs were analyzed using the institutional central laboratory blood gas analyzer. In the Normoxia Group, ABGs were analyzed using the integrated Quantum system (Spectrum Medical Ltd, UK), which provides continuous blood gas analysis. At the beginning of each procedure, the Quantum system was calibrated and synchronized with a reference arterial blood gas analysis to ensure measurement accuracy and inter-device agreement. Although both systems are routinely validated and calibrated, potential inter-device variability cannot be completely excluded and represents a methodological limitation. Carbon dioxide management during CPB followed an alpha-stat strategy targeting normocapnia (PaCO_2_ 35–45 mmHg) in both groups. PaCO_2_ was assessed at the same predefined intervals as arterial blood gas sampling.

The CPB circuit consisted of a roller pump, crystalloid priming solution, and membrane oxygenator. A goal-directed perfusion strategy was followed in both groups, targeting indexed oxygen delivery (DO_2_i) ≥280 mL/min/m^2^, mean arterial pressure between 60 and 70 mmHg, and pump flows indexed to body surface area. The primary methodological difference between groups involved oxygenation monitoring and titration:

- In the Hyperoxia Group, CPB was managed using the S5 LivaNova heart-lung machine platform with Sechrist gas blender, without continuous PaO_2_/PaCO_2_ monitoring. Adjustments in FiO_2_ and sweep gas flow were guided solely by intermittent ABG measurements, yielding sustained PaO_2_ levels typically >200 mmHg. Only mixed venous oxygen saturation was monitored continuously via in-line optical oximetry integrated into the CPB circuit.
- In the Normoxia Group, CPB was performed using the Quantum Perfusion System with Quantum Ventilation Module by Spectrum Medical Ltd, UK, enabling continuous real-time monitoring and feedback control of PaO_2_/PaCO_2_. This configuration permitted dynamic titration of FiO_2_ and sweep gas flow to maintain PaO_2_ within physiological ranges (90–130 mmHg) during bypass.

In both groups, venous saturation, hematocrit, lactat, and systemic temperature were continuously monitored, and pump flow was adjusted to ensure DO_2_i targets were met. Lactate concentration was measured intermittently through arterial blood gas sampling at predefined time points; no continuous lactate sensor was used.

### Data collection and outcome measures

Intraoperative perfusion-related variables, including PaO_2_, mixed venous oxygen saturation (SvO_2_), indexed oxygen extraction ratio, regional cerebral oxygen saturation measured by near-infrared spectroscopy (rSO_2_). Baseline arterial lactate concentration was recorded after induction of anesthesia and before initiation of CPB and lactate concentration, were recorded at predefined intervals during CPB. Perioperative laboratory measurements included serial cardiac troponin levels and oxidative stress biomarkers (hypoxia-inducible factor-1α, total oxidant status, total antioxidant status, and oxidative stress index) collected preoperatively, during CPB, and at 6, 12, and 24 postoperative hours. Clinical outcomes included postoperative left ventricular ejection fraction, requirement for inotropic support following CPB weaning, duration of mechanical ventilation, and length of intensive care unit and hospital stay. Exploratory outcomes comprised lactate kinetics at 0, 6, 12, and 24 h, acute kidney injury according to KDIGO criteria, new-onset atrial fibrillation within 48 h postoperatively, and major adverse cardiac events (MACE) within 30 days.

### Statistical analysis

Statistical analysis was conducted using IBM SPSS Statistics version v29.0 (IBM Corp., Armonk, NY, USA). Continuous variables were tested for normality using the Shapiro–Wilk test and presented as mean ± standard deviation or median with interquartile range. Group comparisons were performed using independent-sample Student’s t test or Mann–Whitney U test as appropriate. Repeated measures across perioperative time points (PaO_2_, SvO_2_, lactate, troponin, oxidative stress biomarkers) were analyzed using repeated-measures ANOVA or mixed-effects linear models with Bonferroni correction for multiple comparisons. Categorical data were compared using chi-square or Fisher’s exact test. Logistic regression models were applied to evaluate associations between oxygenation strategy and postoperative complications, adjusting for relevant clinical covariates when appropriate. A two-sided *p*-value <0.05 was considered statistically significant. An a priori power analysis was performed to assess the adequacy of the sample size. Based on previously reported differences in perioperative troponin release and postoperative left ventricular function, a sample size of 25 patients per group was estimated to provide ≥80% statistical power to detect clinically meaningful between-group differences.

## Results

A total of 50 patients were included in the analysis, equally distributed between the Normoxia and Hyperoxia groups. Baseline demographic characteristics, anthropometric parameters, and operative variables were well balanced between groups, as summarized in (Table 1). No significant differences were observed in age, sex distribution, body surface area, preoperative left ventricular ejection fraction, cardiopulmonary bypass duration, or aortic cross-clamp time, indicating a high degree of comparability between cohorts. Baseline arterial lactate concentrations were comparable between groups (1.22 ± 0.28 vs 1.25 ± 0.30 mg/dL, *p* = 0.71), confirming similar preoperative metabolic status. All patients received a standardized myocardial protection strategy based on intermittent isothermal blood cardioplegia. Each patient of both groups underwent three cardioplegia administrations, consisting of one induction dose and two maintenance doses. The induction dose was delivered over a mean duration of 3 min, with a mean volume of 620 ± 110 mL, while each maintenance dose was administered over 2 min, with a mean volume of 510 ± 89 mL. During the aortic cross-clamp period, the myocardial protection protocol resulted in three ischemic intervals and two controlled intraclamp reperfusion phases associated with cardioplegia delivery, followed by one final global reperfusion at aortic declamping. The cardioplegia protocol and timing were consistent across both study groups. During cardiopulmonary bypass, indexed oxygen delivery was maintained above the predefined goal-directed perfusion threshold in both groups and remained comparable throughout the procedure. Nevertheless, marked differences emerged in oxygenation-related physiological parameters (Table 2) (Figure 1). Arterial PaCO_2_ values remained within the predefined normocapnic target range (35–45 mmHg) throughout CPB in both groups and did not differ significantly at any time point (Table 2), indicating comparable carbon dioxide management during bypass. The Hyperoxia Group showed significantly higher arterial oxygen tension and mixed venous oxygen saturation at all measured time points, whereas the Normoxia Group maintained PaO_2_ within the targeted physiological range (Figure 2). Despite similar systemic oxygen delivery and regional cerebral oxygen saturation measured by near-infrared spectroscopy (rSO_2_), the Hyperoxia Group exhibited a consistently lower indexed oxygen extraction ratio and high mixed venous oxygen saturation, reflecting reduced efficiency of oxygen utilization (Figure 3). This was accompanied by significantly higher lactate concentrations during bypass, suggesting a state of relative metabolic inefficiency under hyperoxic conditions. Mean arterial pressure remained similar between groups, excluding hemodynamic instability as a confounding factor. Perioperative laboratory analyses revealed no significant differences in hemoglobin levels, hematocrit, platelet count, or renal function markers across all time points (Table 3). In contrast, biomarkers of myocardial injury differed substantially between oxygenation strategies. Cardiac troponin levels were significantly lower in the Normoxia Group during CPB and at 6, 12, and 24 h postoperatively, indicating reduced myocardial ischemia–reperfusion injury compared with the Hyperoxia Group. Markers of oxidative stress and hypoxia-related signaling further differentiated the two strategies (Table 4). Patients managed with hyperoxic oxygenation showed significantly higher intraoperative and postoperative levels of hypoxia-inducible factor-1α, total oxidant status, and oxidative stress index, along with a concomitant reduction in total antioxidant capacity. Conversely, the Normoxia Group demonstrated a more attenuated oxidative response and better preservation of antioxidant defenses throughout the perioperative period. These physiological and biochemical differences translated into clinically meaningful early postoperative outcomes. As shown in Table 1, patients in the Normoxia Group exhibited higher postoperative left ventricular ejection fraction assessed by transesophageal echocardiography, and a significantly reduced need for inotropic support with dobutamine following separation from cardiopulmonary bypass. Dobutamine was used as first-line inotropic support in accordance with current ESC/EACTS guidelines and clinical practice on myocardial revascularization.^12–14^ In the Normoxia Group, dobutamine was required in 6 patients (24%) and was administered at a mean dose of 3.6 ± 0.9 µg/kg/min (dilution: 250 mg in 250 mL saline) for a mean duration of 14 ± 5 h following separation from cardiopulmonary bypass. In the Hyperoxia Group, dobutamine support was required in 14 patients (56%) and was administered at a mean dose of 4.8 ± 1.2 µg/kg/min (dilution: 250 mg in 250 mL saline) for a mean duration of 22 ± 7 h after cardiopulmonary bypass. In addition, intensive care unit stay and overall hospital length of stay were significantly shorter in patients managed with normoxia (Figure 4). Duration of mechanical ventilation was comparable between groups. No patient in either group required postoperative mechanical circulatory support, including intra-aortic balloon pump (IABP) or extracorporeal membrane oxygenation (ECMO). Exploratory outcomes, including acute kidney injury, postoperative atrial fibrillation, and 30-days major adverse cardiac events, did not differ significantly, although trends consistently favored the Normoxia Group.

**Table 2.** Cardiopulmonary bypass trend parameters Normoxia group versus Hyperoxia group.

Parameter	Time point (min during CPB)	Normoxia group (mean ± SD) (*n* = 25)	Hyperoxia group (mean ± SD) (*n* = 25)	*p* value
Indexed oxygen delivery (ml/min/m^2^)	10	336.8 ± 22.4	329.6 ± 39.5	0.41
	20	340.9 ± 23.1	331.8 ± 38.1	0.33
	30	344.6 ± 24.8	334.9 ± 36.7	0.29
	40	348.1 ± 25.2	337.3 ± 35.4	0.25
	50	351.2 ± 26.6	340.8 ± 34.9	0.22
	60	357.6 ± 27.9	346.7 ± 33.8	0.20
	70	360.9 ± 28.7	349.9 ± 33.1	0.19
Arterial partial pressure of oxygen (mmHg)	10	162.4 ± 21.6	276.8 ± 40.9	0.018
	20	104.7 ± 2.1	264.9 ± 39.2	0.019
	30	108.9 ± 3.4	252.6 ± 37.8	0.021
	40	103.5 ± 1.7	241.3 ± 35.9	0.024
	50	109.2 ± 2.1	231.8 ± 34.6	0.028
	60	101.6 ± 0.4	214.7 ± 32.8	0.006
	70	107.9 ± 1.9	205.6 ± 31.5	0.008
Arterial partial pressure of carbon dioxide (mmHg)	10	42.3 ± 2.1	42.1 ± 2.2	0.74
	20	42.6 ± 2.0	42.4 ± 2.1	0.71
	30	42.8 ± 2.0	42.6 ± 2.0	0.69
	40	43.1 ± 1.9	42.9 ± 2.0	0.67
	50	43.0 ± 1.8	42.8 ± 1.9	0.65
	60	42.7 ± 1.9	42.6 ± 2.0	0.83
	70	42.5 ± 2.0	42.4 ± 2.1	0.86
Mean arterial pressure (mmHg)	10	61.8 ± 4.9	59.4 ± 5.3	0.19
	20	63.2 ± 5.1	60.8 ± 5.5	0.20
	30	64.6 ± 5.4	62.1 ± 5.7	0.18
	40	65.8 ± 5.6	63.4 ± 5.8	0.21
	50	66.9 ± 5.7	64.6 ± 6.0	0.24
	60	68.6 ± 5.9	66.7 ± 6.2	0.31
	70	69.4 ± 6.0	67.8 ± 6.3	0.37
Mixed venous saturation (%)	10	72.9 ± 5.5	80.1 ± 2.4	0.041
	20	72.4 ± 5.7	79.4 ± 2.6	0.038
	30	72.1 ± 5.6	78.6 ± 2.8	0.044
	40	72.3 ± 5.4	78.0 ± 2.9	0.047
	50	72.8 ± 5.2	77.5 ± 3.1	0.049
	60	73.6 ± 5.0	76.9 ± 3.2	0.07
	70	74.1 ± 4.8	76.5 ± 3.3	0.11
Indexed Oxygen Extraction Ratio (O_2_ERi)	10	28.1 ± 5.0	20.6 ± 2.1	0.028
	20	28.6 ± 5.2	20.9 ± 2.2	0.024
	30	28.9 ± 5.1	21.2 ± 2.3	0.021
	40	28.3 ± 4.9	21.5 ± 2.4	0.027
	50	27.6 ± 4.7	21.8 ± 2.5	0.031
	60	26.3 ± 4.4	22.4 ± 2.8	0.08
	70	25.7 ± 4.2	22.7 ± 2.9	0.12
Lactate (mg/dL)	10	1.34 ± 0.32	2.76 ± 0.56	0.017
	20	1.30 ± 0.31	2.60 ± 0.52	0.018
	30	1.26 ± 0.30	2.41 ± 0.48	0.020
	40	1.22 ± 0.29	2.25 ± 0.46	0.022
	50	1.18 ± 0.28	2.08 ± 0.44	0.026
	60	1.12 ± 0.27	1.78 ± 0.40	0.033
	70	1.09 ± 0.26	1.65 ± 0.38	0.041
Cerebral NIRS (rSO_2_, %)	10	66.8 ± 5.4	67.5 ± 5.2	0.62
	20	65.9 ± 5.6	66.7 ± 5.5	0.58
	30	65.4 ± 5.7	66.1 ± 5.6	0.61
	40	65.6 ± 5.5	66.3 ± 5.4	0.59
	50	65.8 ± 5.3	66.5 ± 5.2	0.57
	60	66.2 ± 5.1	66.9 ± 5.0	0.55
	70	66.5 ± 4.9	67.1 ± 4.8	0.53

NIRS, near-infrared spectroscopy; rSO_2_, regional cerebral oxygen saturation measured by near-infrared spectroscopy. O_2_ERi, indexed oxygen extraction ratio; CPB, cardiopulmonary bypass.

**Figure 1.**
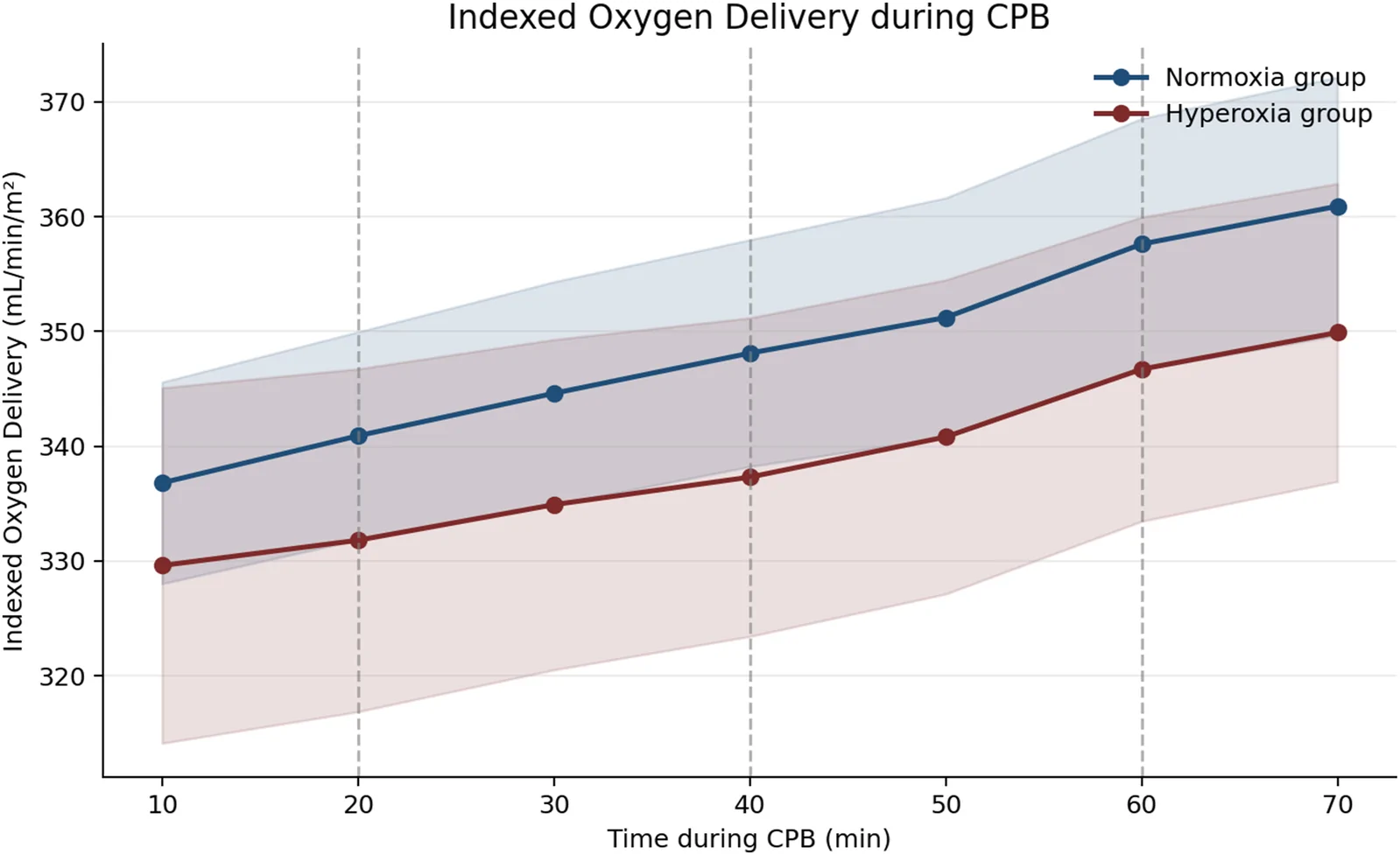
Indexed oxygen delivery during cardiopulmonary bypass (Normoxia group vs Hyperoxia group). Time course of indexed oxygen delivery (DO₂i) during cardiopulmonary bypass in the Normoxia group and Hyperoxia group. Data are presented as mean values with 95% confidence intervals. Vertical dashed lines indicate the timing of intermittent isothermal blood cardioplegia administration at 20-min intervals. Indexed oxygen delivery remained comparable between groups throughout cardiopulmonary bypass.

**Figure 2.**
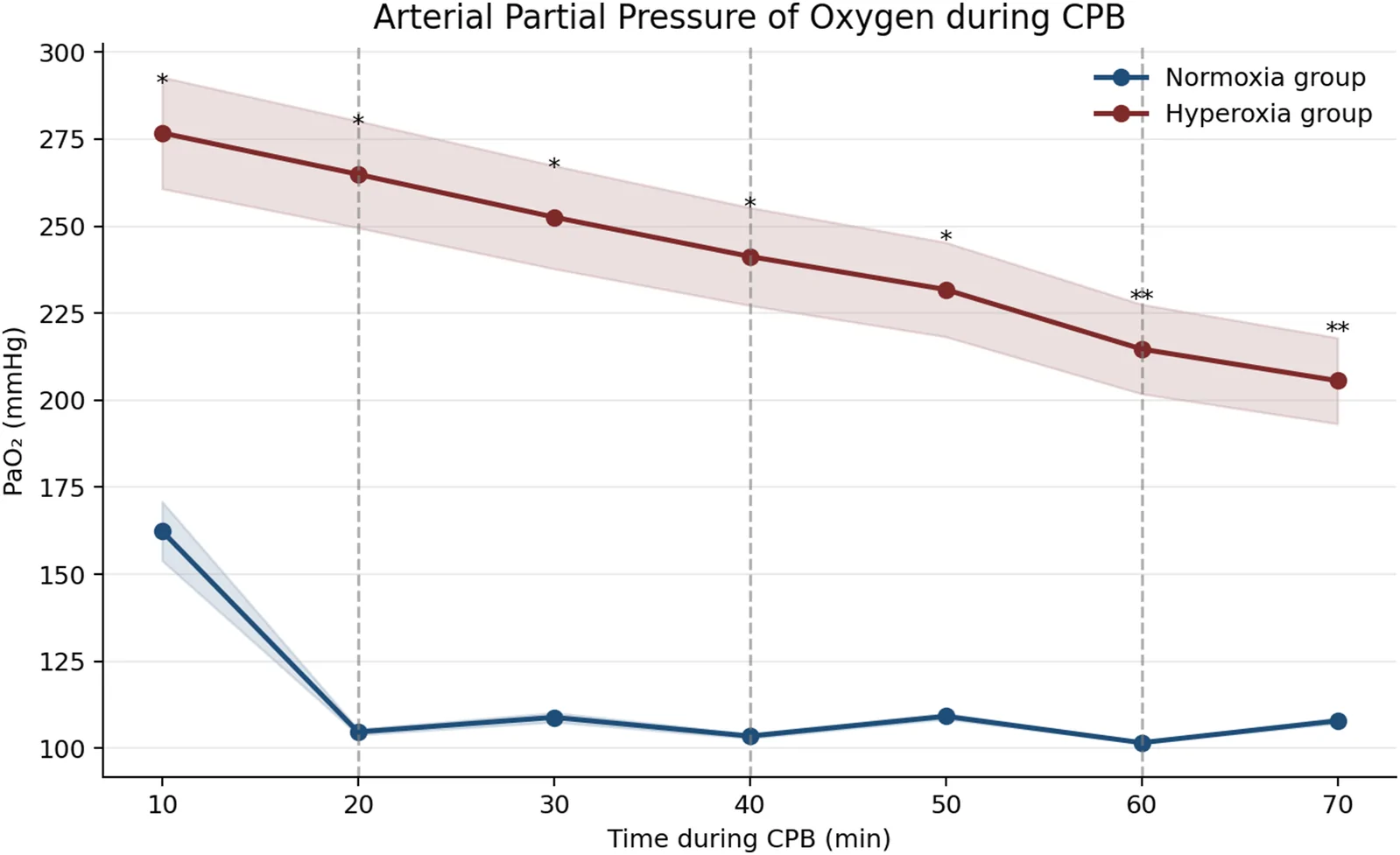
Arterial partial pressure of oxygen during cardiopulmonary bypass (Normoxia group vs Hyperoxia group). Arterial partial pressure of oxygen (PaO₂) measured at predefined time points during cardiopulmonary bypass in the Normoxia group and Hyperoxia group. Values are expressed as mean values with 95% confidence intervals. Vertical dashed lines represent cardioplegia delivery every 20 min. PaO₂ was consistently and significantly higher in the Hyperoxia group compared with the Normoxia group at multiple time points (*p* < 0.05, *p* < 0.01).

**Figure 3.**
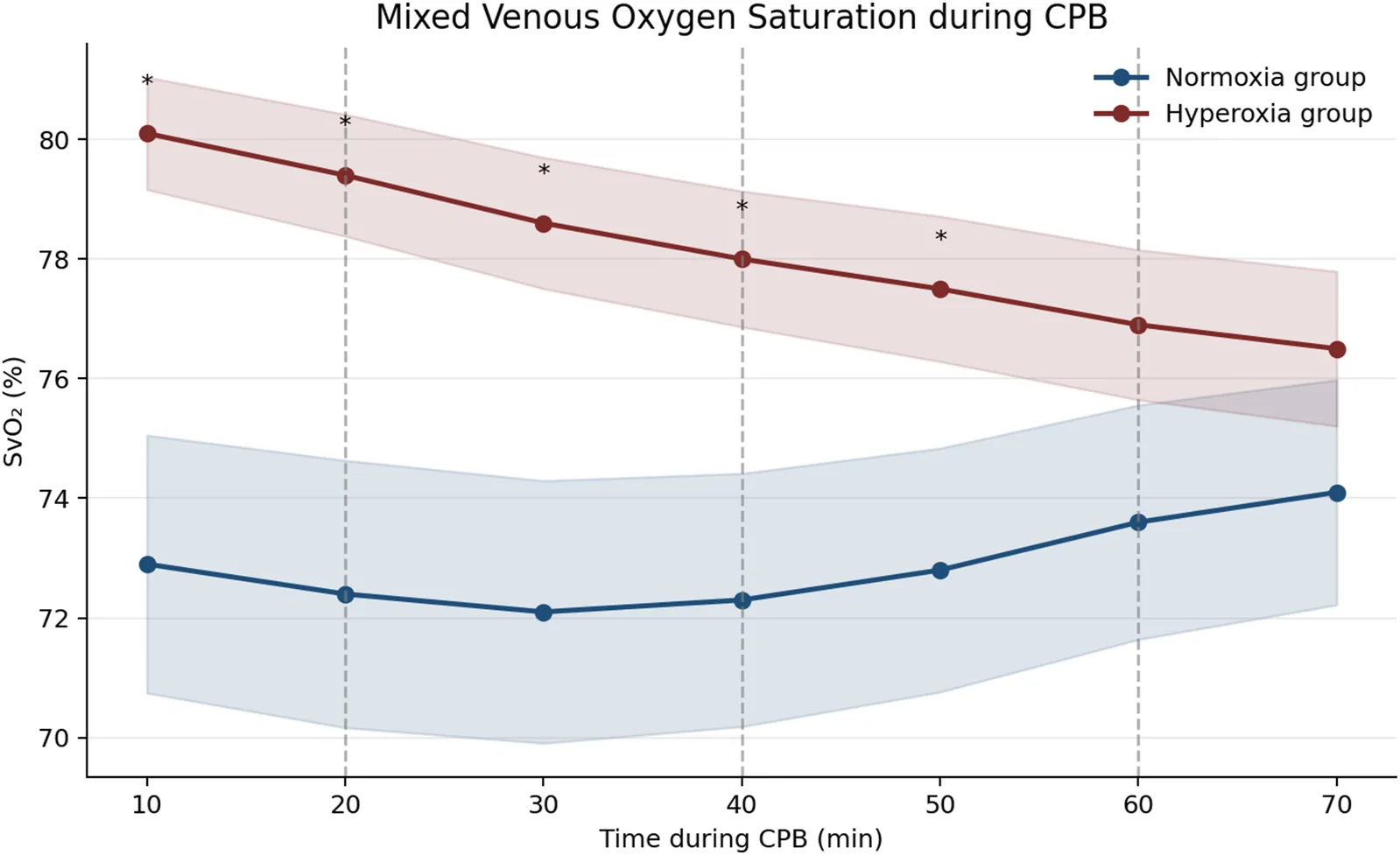
Mixed venous oxygen saturation during cardiopulmonary bypass (Normoxia group vs Hyperoxia group). Trends in mixed venous oxygen saturation (SvO₂) during cardiopulmonary bypass in the Normoxia group and Hyperoxia group. Data are shown as mean values with 95% confidence intervals. Vertical dashed lines denote intermittent cardioplegia administration at 20-min intervals. The Hyperoxia group exhibited higher SvO₂ values during early cardiopulmonary bypass, reflecting reduced oxygen extraction compared with the Normoxia group (*p* < 0.05).

**Table 3.** Laboratory test Normoxia group versus Hyperoxia group.

Parameter	Time point	Normoxia group (mean ± std dev) (*n* = 25)	Hyperoxia group (mean ± std dev)	*p*-value
Hemoglobin	PreOp	11.24 ± 1.15	11.51 ± 1.35	0.69
	Pump	8.21 ± 1.22	8.19 ± 1.07	0.73
	Post 6 h	9.89 ± 0.94	9.46 ± 1.18	0.65
	Post 12 h	9.53 ± 1.19	9.52 ± 1.21	0.95
	Post 24 h	9.02 ± 0.2	8.81 ± 0.16	0.44
Hematocrit (%)	PreOp	33.54 ± 3.27	32.88 ± 3.266	0.48
	Pump	27.14 ± 3.19	27.22 ± 2.9	0.62
	Post 6 h	29.81 ± 2.4	28.97 ± 3.35	0.54
	Post 12 h	27.6 ± 2.3	27.98 ± 3.1	0.49
	Post 24 h	26.51 ± 2.9	26.17 ± 2.22	0.24
Platelets	PreOp	199.17 ± 27.17	186.11 ± 20.3	0.35
	Pump	179.2 ± 46.8	173.24 ± 36.44	0.59
	Post 6 h	208 ± 23	199.14 ± 46,11	0.32
	Post 12 h	188 ± 67	182.23 ± 51.04	0.34
	Post 24 h	179 ± 43	180.23 ± 33.41	0.84
Creatinin	PreOp	0.89 ± 0.19	0.91 ± 0.17	0.54
	Pump	0.80 ± 0.18	0.84 ± 0.22	0.88
	Post 6 h	1.09 ± 0.1	1.11 ± 0.22	0.43
	Post 12 h	1.05 ± 0.3	1.03 ± 0.31	0.76
	Post 24 h	1.03 ± 0.4	1.02 ± 0.49	0.78
Troponin	PreOp	0.01 ± 0.00	0.03 ± 0.11	0.67
	Pump	0.44 ± 0.55	1.45 ± 0.18	0.04
	Post 6 h	1.27 ± 0.6	3.02 ± 0.16	0.04
	Post 12 h	1.2 ± 0.1	4.61 ± 5.21	0.049
	Post 24 h	1.6 ± 0.3	3.49 ± 0.52	0.002

**Table 4.** Perioperative effects of Normoxia versus Hyperoxia and oxidative stress biomarkers.

Biomarker	Time point	Normoxia group (mean ± std dev) (*n* = 25)	Hyperoxia group (mean ± std dev)	*p*. value
HIF-1α	Preoperative	0.74 ± 0.35	0.68 ± 0.32	0.61
	On pump	2.62 ± 1.02	4.18 ± 1.08	0.004
	6 h postoperative	6.21 ± 2.14	8.94 ± 3.01	0.003
	12 h postoperative	4.08 ± 1.57	5.76 ± 1.71	0.006
	24 h postoperative	2.71 ± 0.93	4.42 ± 1.02	0.012
Total oxidant status	Preoperative	11.98 ± 3.24	13.86 ± 2.31	0.29
	On pump	21.63 ± 5.87	32.41 ± 6.14	0.007
	6 h postoperative	27.94 ± 6.21	39.88 ± 5.76	0.004
	12 h postoperative	17.92 ± 6.03	27.61 ± 5.44	0.009
	24 h postoperative	12.89 ± 3.42	22.74 ± 3.68	0.005
Total antioxidant status	Preoperative	0.75 ± 0.15	0.71 ± 0.14	0.58
	On pump	0.56 ± 0.18	0.39 ± 0.16	0.021
	6 h postoperative	0.46 ± 0.15	0.26 ± 0.09	0.003
	12 h postoperative	0.49 ± 0.12	0.31 ± 0.08	0.004
	24 h postoperative	0.58 ± 0.13	0.37 ± 0.11	0.006
Oxidative stress index	Preoperative	17.46 ± 7.02	20.11 ± 7.48	0.55
	On pump	49.87 ± 28.94	92.63 ± 39.82	0.002
	6 h postoperative	74.21 ± 30.17	151.84 ± 46.53	<0.001
	12 h postoperative	41.72 ± 18.46	88.95 ± 31.84	0.001
	24 h postoperative	25.36 ± 8.41	62.78 ± 23.96	0.002

**Figure 4.**
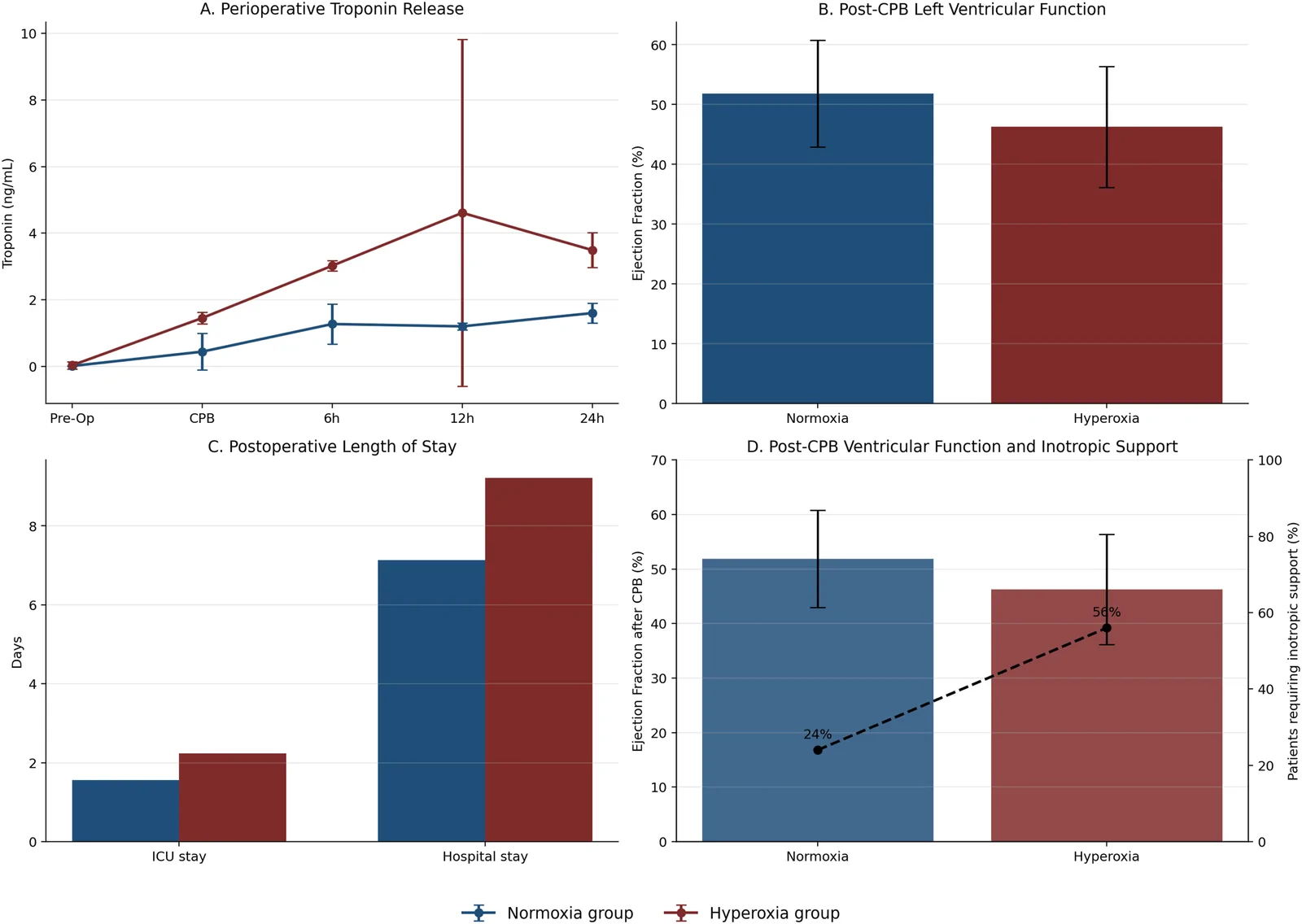
Perioperative myocardial injury, cardiac function, and clinical outcomes (Normoxia group vs Hyperoxia group).

## Discussion

The present study demonstrates that arterial blood oxygenation management during CPB is a relevant determinant of perfusion efficiency, myocardial injury, and oxidative stress in patients undergoing elective CABG. By comparing two consecutive cohorts managed with distinct oxygenation strategies, otherwise identical from the surgical, anesthetic, perfusion, and myocardial protection protocols, we were able to highlight the physiological consequences of hyperoxic versus physiological oxygen delivery during CPB. A principal finding is that supra-physiological PaO_2_ levels were associated with impaired oxygen utilization rather than improved tissue oxygenation. Despite comparable indexed oxygen delivery, hyperoxic patients exhibited higher mixed venous oxygen saturation, lower oxygen extraction ratios, and increased lactate concentrations, indicating reduced metabolic efficiency. These findings are consistent with functional microvascular shunting and suggest that excessive arterial oxygen tension does not translate into effective cellular oxygen uptake and enhanced metabolism.^1,2^ Importantly, PaCO_2_ levels were tightly controlled and comparable between groups throughout CPB. Therefore, the observed differences in oxygen extraction ratio, lactate kinetics, myocardial injury markers, and oxidative stress are unlikely to be confounded by CO_2_-mediated vasoreactivity or Bohr-effect–related shifts in oxygen dissociation.^3,4^ Previous studies investigating restrictive versus liberal oxygen strategies during cardiac surgery have yielded conflicting results.^3,6,7,15^ Notably, the randomized trial by Wiberg et al. comparing FiO_2_ 50% versus 100% during and after CPB did not demonstrate a clear difference in major adverse outcomes.^10^ However, in these studies, true physiological normoxia was never actively achieved, as oxygenation was guided by fixed FiO_2_ settings and intermittent arterial blood gas analysis rather than continuous PaO_2_ monitoring. Consequently, patients assigned to restrictive strategies were still frequently exposed to supra-physiological PaO_2_ levels, particularly during reperfusion, potentially attenuating between-group differences.^15^ All patients received intermittent isothermal blood cardioplegia, which may have influenced the magnitude of ischemia–reperfusion injury observed. Blood cardioplegia provides oxygen-carrying capacity and intrinsic antioxidant buffering compared with crystalloid solutions. It is conceivable that the differences between normoxic and hyperoxic reperfusion strategies might have been attenuated or amplified under crystalloid cardioplegia; however, this hypothesis cannot be addressed within the present study and warrants further investigation.^7^ Microcirculatory dysfunction provides a unifying pathophysiological explanation for our findings. Hyperoxia induces arteriolar and capillary vasoconstriction, leading to heterogeneous perfusion and maldistribution of blood flow.^5^ Experimental and clinical studies have shown that hyperoxia reduces coronary and cerebral blood flow despite increased arterial oxygen content, largely through oxidative stress–mediated alterations in vascular tone.^6^ Emerging evidence further indicates that hyperoxia impairs endothelium-independent vasodilation via oxidative modification of soluble guanylate cyclase, reducing nitric oxide signaling and vascular smooth muscle relaxation.^7^ These mechanisms are particularly relevant during myocardial ischemia–reperfusion. In our protocol, the myocardium was exposed to repeated ischemic intervals and controlled intraclamp reperfusion phases associated with cardioplegia delivery, followed by final global reperfusion.^9,11,16^ Hyperoxia during these phases likely amplifies reactive oxygen species generation, mitochondrial dysfunction, and microvascular impairment. Consistent with this hypothesis, hyperoxic management was associated with higher postoperative troponin release, reduced early postoperative left ventricular function, and increased need for inotropic support despite apparently similar CABG procedures and results. From a perfusion management perspective, our results highlight an important gap in current goal-directed perfusion (GDP) strategies. While GDP has focused primarily on maintaining adequate indexed oxygen delivery (DO_2i_) > 280 ml/min/m^2^ to reduce acute kidney injury,^8^ the specific role of normoxia versus hyperoxia during ischemia and reperfusion phases, particularly at the myocardial level, has not been adequately addressed. Oxygen tension is often treated as a safety margin rather than a titratable physiological variable. Continuous real-time PaO_2_ and PaCO_2_ monitoring allows oxygen therapy to be integrated into GDP frameworks, minimizing unrecognized hyperoxic exposure during critical phases of CPB. This study has some limitations, including its retrospective design and modest patient sample size, as well as the lack of direct assessment of microcirculatory flow and inflammatory markers.^12,13,17^ Although PaCO_2_ was comparable between groups, subtle regional microcirculatory effects of CO_2_ cannot be completely excluded and warrant further mechanistic investigation. Nonetheless, the concordance between perfusion parameters, metabolic indices, oxidative stress markers, myocardial injury biomarkers, and early clinical outcomes strengthens the internal validity of our findings.^14^ An additional methodological limitation deserves consideration. Arterial blood gas analyses were performed using two different monitoring platforms across the study periods: a conventional laboratory blood gas analyzer in the Hyperoxia group and an integrated continuous monitoring system in the Normoxia group. Although both systems are routinely validated and calibrated according to manufacturer specifications, and the Quantum system was synchronized with a reference arterial blood gas analysis at the beginning of each procedure, residual inter-device measurement variability cannot be completely excluded. Such variability may have influenced absolute PaO_2_ values; however, the magnitude and consistency of between-group differences across multiple physiological, biochemical, and clinical parameters make it unlikely that measurement variability alone explains the observed findings.

## Conclusions

In this retrospective study of patients undergoing elective coronary artery bypass grafting with cardiopulmonary bypass, a normoxia strategy during CPB was associated with more efficient oxygen utilization, reduced oxidative stress, and attenuated perioperative myocardial injury compared with conventional hyperoxic oxygenation, despite similar indexed oxygen delivery. Patients managed with physiological PaO_2_ targets exhibited improved early postoperative ventricular function, reduced need for inotropic support, and shorter intensive care unit and hospital stays. These findings suggest that supraphysiological oxygen tensions during CPB may impair microcirculatory oxygen utilization and exacerbate ischemia–reperfusion injury. Continuous PaO_2_ and PaCO_2_ monitoring enabling physiology-guided oxygen titration may represent a clinically relevant approach to optimizing myocardial protection during cardiac surgery. These observations were obtained in the context of blood cardioplegia–based myocardial protection and should be interpreted accordingly.

## Data Availability

All data supporting the findings of this study are available from the corresponding author upon reasonable request.*

## References

[1] Lopez MG Pandey AK Hennessy C , et al. Effects of oxygen on perioperative vascular function: a randomized clinical trial. J Am Heart Assoc 2025; 14: e041778. 10.1161/JAHA.125.041778 PMC12450019 40673571

[2] Lopez MG Pretorius M Shotwell MS , et al. The risk of oxygen during cardiac surgery (ROCS) trial: study protocol for a randomized clinical trial. Trials 2017; 18: 295. 10.1186/s13063-017-2021-5 PMC5485572 28651648

[3] Heinrichs J Lodewyks C Neilson C , et al. The impact of hyperoxia on outcomes after cardiac surgery: a systematic review and narrative synthesis. Can J Anaesth 2018; 65: 923–935. 10.1007/s12630-018-1143-x 29721817

[4] Shaefi S Shankar P Mueller AL , et al. Intraoperative oxygen concentration and neurocognition after cardiac surgery. Anesthesiology 2021; 134: 189–201. 10.1097/ALN.0000000000003650 PMC7855826 33331902

[5] Smit B Smulders YM de Waard MC , et al. Moderate hyperoxic versus near-physiological oxygen targets during and after coronary artery bypass surgery: a randomised controlled trial. Crit Care 2016; 20: 55. 10.1186/s13054-016-1240-6 PMC4788916 26968380

[6] Boisramé-Helms J Radermacher P Asfar P . Cardiac surgery, a right target for hyperoxia? Crit Care 2016; 20: 162. 10.1186/s13054-016-1347-9 PMC4910213 27306619

[7] Spoelstra-de Man AM Smit B Oudemans-van Straaten HM , et al. Cardiovascular effects of hyperoxia during and after cardiac surgery. Anaesthesia 2015; 70: 1307–1319. 10.1111/anae.13218 26348878

[8] Ranucci M Johnson I Willcox T , et al. Goal-directed perfusion to reduce acute kidney injury: a randomized trial. J Thorac Cardiovasc Surg 2018; 156: 1918–1927.e2. 10.1016/j.jtcvs.2018.04.045 29778331

[9] Condello I Federico M Condello S . The dynamic interplay between endothelial reactivity, temperature, and vascular resistance in cardiopulmonary bypass. Ann Med Surg 2025; 87: 4695–4700. 10.1097/MS9.0000000000003481 PMC12333845 40787570

[10] Wiberg S Møller CH Kjaergaard J , et al. Restrictive versus liberal oxygenation in patients undergoing cardiopulmonary bypass-assisted heart surgery: a randomised controlled trial. Br J Anaesth 2025; 135: 1618–1625. 10.1016/j.bja.2025.08.005 PMC12799424 40975689

[11] Böning A Menzebach S Heep M , et al. Calafiore cardioplegia offers better myocardial protection in adult and senescent rat hearts than Del Nido cardioplegia. Perfusion 2024; 39: 1605–1613. 10.1177/02676591231204290 37740272

[12] Amin S Werner RS Madsen PL , et al. Intraoperative bypass graft flow measurement with transit time flowmetry: a clinical assessment. Ann Thorac Surg 2018; 106: 532–538. 10.1016/j.athoracsur.2018.02.067 29605596

[13] Byrne RA Rossello X Coughlan JJ , et al. 2023 ESC guidelines for the management of acute coronary syndromes. Eur Heart J 2023; 44: 3720–3826. 10.1093/eurheartj/ehad191 37622654

[14] Coskun C Borulu F Emir I , et al. Investigation of the relationship between cerebral near-infrared spectroscopy measurements and cerebrovascular event in coronary artery bypass grafting operation. Braz J Cardiovasc Surg 2020; 35: 465–470. 10.21470/1678-9741-2019-0050 PMC7454614 32864925

[15] Grillo ITC Santana ESP Passos FS , et al. Impact of intraoperative hyperoxia versus normoxia on mortality and organ injury in cardiac surgery: a systematic review and meta-analysis of randomized clinical trials. J Cardiothorac Vasc Anesth 2026; 40: 509–521. 10.1053/j.jvca.2025.09.042 41152119

[16] Murkin JM . Monitoring and optimization of the microcirculation during CPB. J Thorac Dis 2019; 11(Suppl 10): S1489–S1491. 10.21037/jtd.2019.02.100 PMC6586585 31293798

[17] Fujii Y Shirai M Tsuchimochi H , et al. Hyperoxic condition promotes an inflammatory response during cardiopulmonary bypass in a rat model. Artif Organs 2013; 37: 1034–1040. 10.1111/aor.12125 23865518

